# Inactivation of Pathogenic Viruses by Plant-Derived Tannins: Strong Effects of Extracts from Persimmon (*Diospyros kaki*) on a Broad Range of Viruses

**DOI:** 10.1371/journal.pone.0055343

**Published:** 2013-01-25

**Authors:** Kyoko Ueda, Ryoko Kawabata, Takashi Irie, Yoshiaki Nakai, Yukinobu Tohya, Takemasa Sakaguchi

**Affiliations:** 1 Department of Virology, Institute of Biomedical & Health Sciences, Hiroshima University, Hiroshima, Japan; 2 Altan Corporation, Tokyo, Japan; 3 Department of Veterinary Medicine, College of Biosource Sciences, Nihon University, Kanagawa, Japan; College of Medicine, Hallym University, Republic of Korea

## Abstract

Tannins, plant-derived polyphenols and other related compounds, have been utilized for a long time in many fields such as the food industry and manufacturing. In this study, we investigated the anti-viral effects of tannins on 12 different viruses including both enveloped viruses (influenza virus H3N2, H5N3, herpes simplex virus-1, vesicular stomatitis virus, Sendai virus and Newcastle disease virus) and non-enveloped viruses (poliovirus, coxsachievirus, adenovirus, rotavirus, feline calicivirus and mouse norovirus). We found that extracts from persimmon (*Diospyros kaki*), which contains ca. 22% of persimmon tannin, reduced viral infectivity in more than 4-log scale against all of the viruses tested, showing strong anti-viral effects against a broad range of viruses. Other tannins derived from green tea, acacia and gallnuts were effective for some of the viruses, while the coffee extracts were not effective for any of the virus. We then investigated the mechanism of the anti-viral effects of persimmon extracts by using mainly influenza virus. Persimmon extracts were effective within 30 seconds at a concentration of 0.25% and inhibited attachment of the virus to cells. Pretreatment of cells with the persimmon extracts before virus infection or post-treatment after virus infection did not inhibit virus replication. Protein aggregation seems to be a fundamental mechanism underlying the anti-viral effect of persimmon tannin, since viral proteins formed aggregates when purified virions were treated with the persimmon extracts and since the anti-viral effect was competitively inhibited by a non-specific protein, bovine serum albumin. Considering that persimmon tannin is a food supplement, it has a potential to be utilized as a safe and highly effective anti-viral reagent against pathogenic viruses.

## Introduction

Animal viruses, which are causative agents of human and animal diseases, can be divided into two types based on their physical properties: enveloped virus, which has a viral envelope composed of a lipid bilayer on its surface, and non-enveloped virus, which lacks an envelope with its protein shell usually being exposed to the environment [1], [2].

Influenza virus, an enveloped virus, is a pathogen that causes respiratory infection. Outbreaks of influenza virus infection occur every year and sometimes cause fatal diseases including pneumonia, secondary bacterial pneumonia and encephalopathy [3]. In 2009, a novel swine-derived influenza virus emerged and caused a worldwide pandemic. Influenza virus is sensitive to detergents or ethanol-based reagents. Human norovirus, a non-enveloped virus, is a causative agent of acute gastroenteritis, affecting many people all over the world [4]. Since a cell culture system for human norovirus has not been fully established, feline calicivirus and mouse norovirus are used as surrogates of human norovirus in anti-virus tests. Human norovirus is generally resistant to usual detergents as well as ethanol-based reagents, causing difficulty in sanitation [4].

Tannins are basically derived from plants and defined as “a material which produces leather from hide” [5]. Tannins prevent plants from being edible for worms and confer resistance to microbes. They have been exploited as food and medicine for their effects against tumors, oxidants or microbes [6]. Tannins include hydrolyzable tannins, which can be hydrolyzed to glucose and gallic acid, and proanthocyanidins or condensed tannins, which are compounds composed of flavonoids. Persimmon tannins, which are condensed tannins from persimmon (*Diospyros kaki*), have been widely utilized for anti-septics, folk medicine and a clarifier for brewing Japanese sake. Green tea tannin, which is a hydrolyzable tannin, has been found to restrict the growth of viruses such as influenza virus and herpes simplex virus type 1 [7], [8], reviewed in [6], [9].

In the present study, we investigated the effects of seven extracts and chemical compounds containing tannins against twelve enveloped and non-enveloped viruses, and we found that persimmon extracts alone inactivated all of the viruses. We further tried to determine the mechanism by which persimmon extracts inactivate viruses.

## Materials and Methods

### Reagents

Persimmon extracts (PE) were prepared by serial procedures of squeezing immature green persimmon fruit, clarification with filtration, and freeze-drying. A batch of PE was shown to contain 21.8% of persimmon tannins (tested by the Japan Food Research Laboratories, Tokyo, Japan, with the Folin-Denis method). Pentagalloyl glucose (PGG) was prepared by crushing gallnuts followed by extraction with water, fractionation using an Octa Docyl Silyl column and freeze-drying. Wattle extracts (WE) from an acacia species, coffee extracts (CE, Caffenol P-100 raw coffee bean extracts), propyl galate (PRG, a synthetic compound), and green tea extracts (GTE, Catechin FP95 green tea extracts) were provided by Fuji Chemical Industry Co., Ltd. (Toyama, Japan). Pyrogallol (PYG, a synthetic compound) was purchased from Kishida Chemical Co., Ltd. (Osaka, Japan). These reagents were individually dissolved to make a 1% (w/w) solution in 10% (w/w) ethanol and kept at 4°C in a dark room as stock solutions. Tannin samples, their abbreviations, and the origins and types of tannin are summarized in Table 1.

**Table 1:** Tannins used in this study.

Sample	Abbreviation	Origin	Tannin Type
persimmon extract	PE	extracts from persimmon	condensed
wattle extract	WE	extracts from acacia	condensed
coffee extract	CE	extracts from coffee beans	(pseudo tannin)
green tea extract	GTE	extracts from green tea	hydrolyzable
pentagalloyl glucose	PGG	extracts from gallnuts	hydrolyzable
propyl gallate	PRG	synthetic compound	hydrolyzable
pyrogallol	PYG	synthetic compound	hydrolyzable

Tannin names and their abbreviations in this paper are shown in the table. Origins of tannins and type of tannin, condensed tannin or hydrolyzable tannin, are also shown.

### Cells and viruses

FL cells (HeLa cell contaminant, a gift from S. Takao, originally purchased from ATCC), Vero cells (African green monkey kidney-derived cells, a gift from S. Takao, originally purchased from JCRB Cell Bank), MA104 cells (macaque monkey fetus kidney-derived cells, a gift from M. Kuzuya, described in [10]) and RAW264.7 cells (mouse macrophage-derived cells, purchased from RIKEN Cell Bank) were propagated in Dulbecco's modified Eagle's minimum essential medium (DMEM, Invitrogen) supplement with 10% fetal calf serum (FCS), penicillin G (100 units/ml, Meiji Seika Pharma, Tokyo, Japan) and streptomycin (100 µg/ml, Meiji Seika Pharma). LLC-MK_2_ cells (macaque monkey kidney-derived cells, described in [11]) and CRFK cells (feline kidney-derived cells, a gift from M. Noda, originally purchased from JCRB Cell Bank) were propagated in Eagle's minimum essential medium (MEM, Invitrogen) supplemented with 10% FCS, penicillin G and streptomycin. MDCK(+) cells (canine kidney-derived cells described in [12]) were propagated in MEM supplemented with 5% FCS, 5% newborn calf serum, penicillin G and streptomycin.

Viruses used in this study are summarized in Table 2. Six enveloped viruses [human influenza virus A/Udorn/72 (H3N2), avian influenza virus A/swan/Shimane/499/83 (H5N3), herpes simplex virus-1, vesicular stomatitis virus, Sendai virus and Newcastle disease virus] and six non-enveloped viruses [poliovirus, Coxsachievirus, adenovirus, rotavirus, feline calicivirus and mouse norovirus] were used. Abbreviations of the viruses, virus families, cells used for anti-virus assay and methods to determine viral infectivity are shown in Table 2. Influenza virus A/swan/Shimane/499/83 (H5N3) was provided by K. Otsuki. Human herpes simplex virus type 1 VR-3, a Vero cell-adapted clinical isolate, was provided by K. Kiyotani. Poliovirus Sabin-1 vaccine strain and coxsachie virus group B type 5 were provided by S. Takao. Feline calicivirus F9 strain was provided by M. Noda. Rotavirus Wa strain was provided by M. Kuzuya. Sendai virus expressing enhanced green fluorescent protein (EGFP) (SeV-EGFP) was generated following the method described in [13].

**Table 2:** Viruses used in this study.

Virus	Abbreviation	Family	Envelope	Cell	Assay Method
A/Udorn/72 (H3N2)	H3N2	*Orthomyxoviridae*	+	MDCK(+)	1*
A/swan/Shimane/499/83 (H5N3)	H5N3	*Orthomyxoviridae*	+	MDCK(+)	1
herpes simplex virus-1, VR-3	HSV	*Herpesviridae*	+	FL	1
vesicular stomatitis virus, New Jersey serotype	VSV	*Rhabdoviridae*	+	FL	1
Sendai virus, Z strain	SeV	*Paramyxoviridae*	+	LLC-MK_2_	2**
Newcastle disease virus, Herts strain	NDV	*Paramyxoviridae*	+	FL	1
poliovirus, Sabin-1	PoV	*Picornaviridae*	−	Vero	1
Coxsachie virus, group B, type 5	CoV	*Picornaviridae*	−	Vero	1
adenovirus type 5, a non-replicating recombinant virus	AdV	*Adenoviridae*	−	FL	2
rotavirus, Wa strain	RoV	*Reoviridae*	−	MA104	2
feline calicivirus, F9 strain	FCV	*Caliciviridae*	−	CRFK	1
mouse norovirus, S7 strain	MNV	*Caliciviridae*	−	RAW264.7	1

Virus name, its abbreviation in this paper, the family that it belongs to, presence(+) or absence(−) of an envelope, cells used for infectivity assay and an assay method for the virus are shown in the table. *1: TCID_50_ method, **2: Immunofluorescent infectious focus assay.

### Cytotoxicity assay

Seven tannin samples were subjected to cytotoxicity assays by using a Cell Proliferation Kit I (Roche Diagnostics, Basel, Switzerland) according to the manufacturer's instructions. Briefly, confluent cells in a 96-well plate were exposed to 50 µl/well of DMEM containing 0.05%, 0.025% or 0.005% of tannins for 24 h in a CO_2_ incubator, the concentration of 0.025% being the same as that used for anti-virus tests in this study. Then 5 μl of MTT (3-[4,5-dimethylth-iazol-2-yl]-2,5-diphenyl tetrazolium bromide) was added and the cells were further incubated for 4 h. The cells were solubilized and optical absorbance at 570 nm was measured by using a microtiter plate reader (Model 680, Bio-Rad Laboratories, Hercules, CA). Cell viability was estimated by comparing values of tannin samples with that of DMEM without tannins.

### Anti-virus assay

Anti-virus assays for H3N2, H5N3, HSV, VSV, NDV, PoV, CoV, FCV and MNV were performed by a standard TCID_50_ method. Briefly, equal volumes of 0.5% tannin solution and virus solution were mixed to make a 0.25% tannin-containing virus solution. After 3-min incubation at room temperature, the solution was 10-fold serially diluted with DMEM. Susceptible cells for a virus in a 96-well plate were inoculated with 50 μl of a diluted virus solution in quadruplicate or octuplicate. After 1-h adsorption, the inoculum was removed and cells were incubated in 100 µl/well of DMEM supplemented with penicillin G and streptomycin. Twenty µg/ml of trypsin was included for the assay of influenza viruses, H3N2 and H5N3, and 5% FCS-containing MEM was used instead of DMEM for the assay of MNV. Water, Dulbecco's phosphate-buffered saline (PBS) and 10% ethanol were used instead of a tannin solution, and the average infectivity of the three was used as virus infectivity of mock-treated control. When the cytopathic effect had fully developed after several days, cells were fixed with ethanol and acetate (5∶1) and further stained with 0.5% amido black 10B in 45% ethanol and 10% acetate. The 50% endpoint of virus infection was determined by the Behrens-Kaeber method, and 50% tissue culture infectious dose (TCID_50_) was calculated. Anti-virus effects were estimated by comparing tannin-treated infectivity with mock-treated infectivity.

Anti-virus assays for SeV, AdV and RoV were performed by an immunofluorescent infectious focus assay. Briefly, tannin-treated and diluted virus solutions were inoculated to susceptible cells on a glass coverslip. After 24 h, cells were fixed and stained using a specific antibody as described previously [14]. Primary antibodies used were rabbit anti-serum for SeV, mouse monoclonal antibody REMI-1 (ARGENE, Varilhes, France) for RoV and mouse monoclonal antibody MAB805 (Merck-Millipore, Darmstadt, Germany) for AdV. Infected cells were counted under a fluorescent microscope (VANOX-T, Olympus, Tokyo, Japan), and virus infectivity was determined and designated as cell-infecting units (CIU)/ml as described previously [11]. Since the virus does not make a cell-to-cell transmission in these combinations of virus and cells, an infected cell represents an infectious focus. (SeV and RoV require exogenous trypsin to spread to neighboring cells and AdV is a replication-incompetent virus lacking the early region of the genome).

Anti-virus effects were recognized when virus infectivity was decreased after tannin treatment by 4 Log_10_ reduction or more (99.99% reduction or more) compared with mock-treated infectivity.

### Effects of PE in steps of virus infection

PE was added in different steps of influenza virus infection in a standard TCID_50_ assay. (1) MDCK cells in a 96-well plate were treated with 100 μl/well of DMEM containing 0.025% PE for 1 h before virus inoculation (pre-infection). (2) Virus solution was mixed with an equal volume of 0.5% PE and incubated for 3 min. After 10-fold serial dilution, 50 μl/well of the diluted virus inoculum was applied to cells in a well and kept at 37°C for 1 h as described above (adsorption). (3) After virus adsorption, the inoculum was replaced with 100 μl/well of DMEM containing 0.025% PE and 20 µg/ml of trypsin (post-infection). After virus infection in the three different conditions, virus infectivity was assessed as described above.

Alternatively, monolayers of LLC-MK_2_ cells on a coverslip in a 35-mm dish were infected with SeV-EGFP at an input multiplicity of infection of 5 under PE treatment in three different conditions, (1) pre-infection, (2) adsorption and (3) post-infection, as described above. After 24 h, EGFP fluorescence was observed under a fluorescent microscope.

### Hemagglutination inhibition (HI) test

HI test was performed according to a standard procedure. Briefly, 25 μl of 0.1% PE or 0.1% bovine serum albumin (BSA) was 2-fold serially diluted in a round-bottom 96-well plate. Influenza virus H3N2 (16 HA in 25 μl) was added to each well and incubated at room temperature for 3 min. Then 0.5% (v/v) of chicken red blood cells in PBS (50 μl) was added to each well and hemagglutination was measured after 1 h.

### SDS-PAGE analysis of PE-treated virus particles

Influenza virus H3N2 particles were concentrated from infected allantoic fluid of chicken eggs by ultracentrifugation, followed by purification in a continuous sucrose gradient in ultracentrifugation and by suspension in PBS as described previously [15]. An equal volume of purified virions (10 μg/μl) or BSA (10 μg/μl) was mixed with 0.5% PE and kept for 3 min. The samples were further mixed with an equal volume of 2× SDS sample buffer (0.5 M Tris-HCl [pH 6.8], 10% sodium dodecyl sulfate (SDS), 20% glycerol, one grain of bromophenol blue, 1.7 M β-mercaptoethanol) and kept at 50°C for 20 min. Proteins were analyzed by 12% SDS-polyacrylamide gel electrophoresis (PAGE) and Coomassie brilliant blue staining as described previously [15].

### Accelerated aging test of persimmon tannin

Accelerated aging was performed according to the guidelines. One percent (w/w) PE in 10% ethanol was kept for 0, 1, 4, or 6 months at 40°C±2°C and at relative humidity of 75%±5% and was subjected to anti-virus test against influenza virus.

## Results

### Cytotoxicity of tannins to cultured cells

Cultured cells used in this study for anti-virus tests, MDCK(+), FL, LLC-MK_2_, Vero, MA104, CRFK and RAW264.7 cells (Table 2), were individually incubated with the seven tannin samples, PE, WE, CE, GTE, PGG, PRG and PYG (Table 1), for 24 h, and viability of the cells was examined by using the MTT assay.

In the anti-viral test, 0.5% tannin sample was mixed with an equal volume of virus solution and then 10-fold serially diluted. Therefore, the highest concentration of tannin to which cells were exposed was 0.025%. We thus employed 0.025% tannin samples together with 0.05% and 0.005% samples.

**Figure 1:**
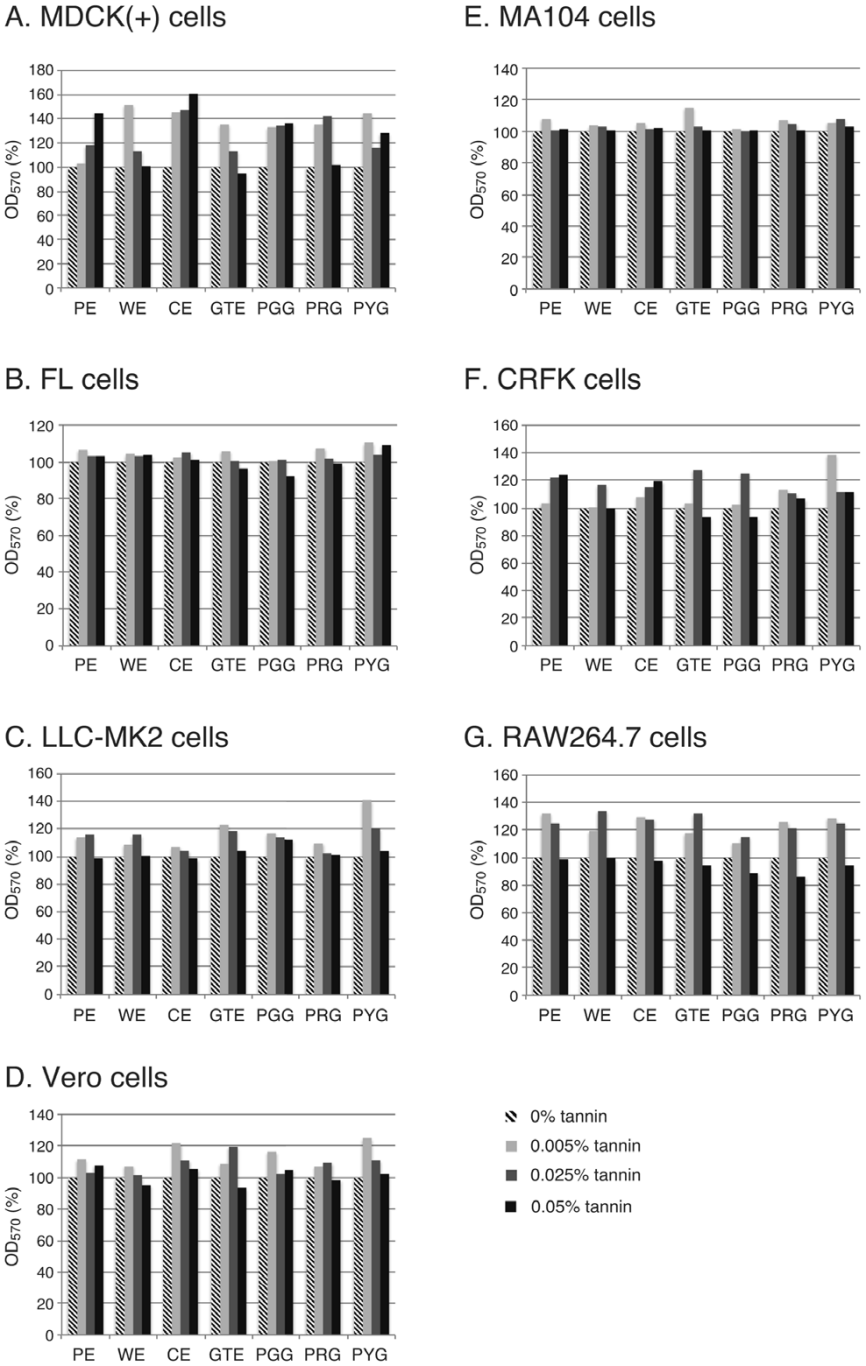
Cytotoxicity of tannins to cultured cells. Seven cultured cell lines, (A) MDCK(+), (B) FL, (C) LLC-MK_2_, (D) Vero, (E) MA104, (F) CRFK and (G) RAW264.7 cells, used for anti-virus tests (Table 2) were individually incubated with 0%, 0.005%, 0.025% or 0.05% of one of the tannin samples (Table 1) in DMEM for 24 h, and viability of the cells was examined by using the MTT assay. The OD_570_ value of 0% tannin was set to 100%, and the OD_570_ values in the presence of tannin samples are shown in percent for each tannin sample.

All of the cells showed more than 100% of MTT staining (OD_570_) in the presence of 0.025% tannin samples compared with 0% tannin-, mock-treated individual cells (Fig. 1). In the presence of 0.05% PRG or 0.05% PGG, MTT staining decreased to ca. 85% in RAW264.7 cells, indicating mild cytotoxicity (Fig. 1). However, no cytotoxicity was observed in other combinations of cells and tannin samples (Fig. 1). It was thus concluded that tannin samples are not cytotoxic in the anti-virus test using 0.5% tannin, which was diluted to a final concentration of 0.025% in the first row of a 96-well plate in the TCID_50_ assay.

### Anti-virus effects of tannins

The abilities of seven different tannin reagents (Table 1) to inactivate viruses were investigated. Five of those tannin reagents were extracts from plants (PE, WE, CE, GTE and PGG) and two were tannin constituents chemically synthesized (PRG and PYG). Six enveloped viruses and six non-enveloped viruses were used for anti-virus assays (Table 2). Enveloped viruses included human and avian influenza viruses (H3N2 and H5N3), herpes simplex virus-1 (HSV), vesicular stomatitis virus (VSV), Sendai virus (SeV) and Newcastle disease virus (NDV), and non-enveloped viruses included poliovirus (PoV), Coxsachie virus (CoV), adenovirus (AdV), rotavirus (RoV), feline calicivirus (FCV), and mouse norovirus (MNV). The last two viruses were used as surrogates of human norovirus. The anti-virus test was performed by measuring virus infectivity after tannin treatment with a standard TCID_50_ method or an immunofluorescent infectious focus method.

**Figure 2:**
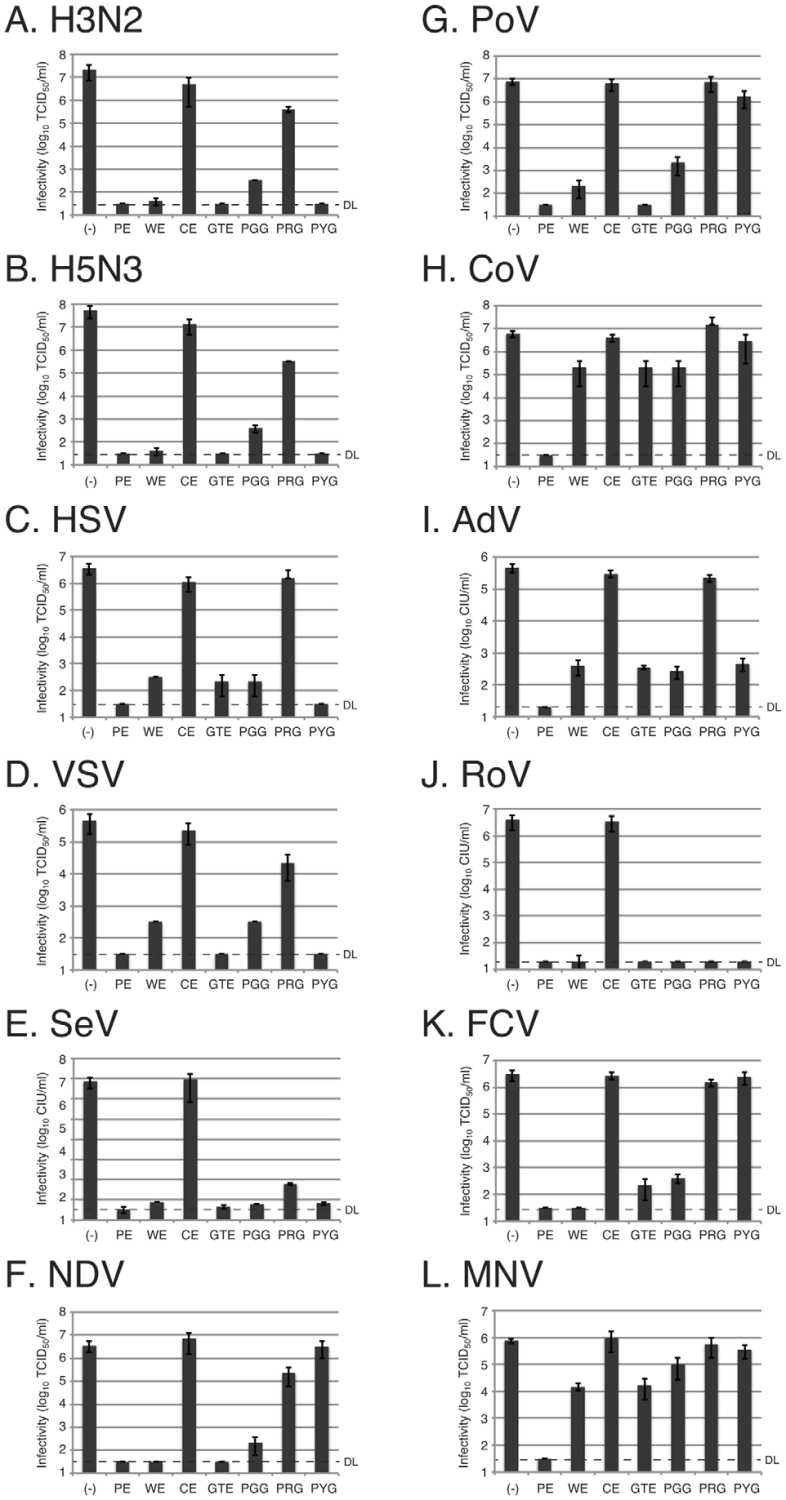
Anti-virus effects of tannins on a broad range of viruses. Each virus was incubated with a 0.5% tannin solution (PE, WE, CE, GTE, PGG, PRG or PYG; summarized in Table 1) for 3 min, and the remaining infectivity was measured by a TCID_50_ method or an immunofluorescent focus assay. Average infectivities of three independent experiments are shown in the graphs and error bars indicate standard deviations. (−), virus was treated with each of water, PBS and 10% ethanol, and the average infectivity of the three is shown as mock-treated control infectivity. A-L, infectivities of twelve different viruses as indicated in the graphs are shown.

The results showed that PE treatment inactivated all of the 12 viruses, suppressing their infectivities to less than the detection limit by more than 4 log reduction (Fig. 2, Table 3). GTE inactivated 9 different viruses, the largest number of viruses next to PE, but did not restrict the infectivity of non-enveloped viruses, AdV, MNV and CoV (Fig. 2, Table 3). WE, PGG, PYG and PRG inactivated 2 to 8 viruses, showing intermediate effects on viruses. In contrast, CE had no suppressive effect on any virus, indicating that it has absolutely no effect on viruses (Fig. 2, Table 3). These results indicate that PE has potent anti-virus effects against a broad range of viruses, including both enveloped and non-enveloped viruses.

**Table 3:** Summary of log_10_ reductions of virus infectivity by tannin treatment.

	Tannins						
Virus	PE	WE	CE	GTE	PGG	PRG	PYG
H3N2	**5.8**	**5.7**	0.7	**5.8**	**4.8**	1.8	**5.8**
H5N3	**6.2**	**6.1**	0.7	**6.2**	**5.1**	2.3	**6.2**
HSV	**5.1**	**4.1**	0.6	**4.2**	**4.2**	0.4	**5.1**
VSV	**4.2**	3.3	0.4	**4.2**	3.3	1.4	**4.2**
SeV	**6.6**	**6.0**	0	**6.2**	**6.1**	**5.1**	**6.0**
NDV	**5.1**	**5.1**	0	**5.1**	**4.2**	1.3	0.06
PoV	**5.4**	**4.5**	0.09	**5.4**	3.6	0.03	0.6
CoV	**5.2**	1.4	0.1	1.5	1.5	0	0.4
AdV	**4.3**	3.2	0.2	3.2	3.3	0.4	3.1
RoV	**5.3**	**5.3**	0.06	**5.3**	**5.3**	**5.3**	**5.3**
FCV	**4.9**	**4.9**	0.05	**4.1**	3.9	0.3	0.1
MNV	**4.3**	1.6	0	1.7	0.9	0.2	0.4

Mean values of log_10_ reduction are shown in the table. Significant reduction, 4 or more, of log_10_ reduction is marked by bold and underline.

### Effective concentration and incubation time of PE

**Figure 3:**
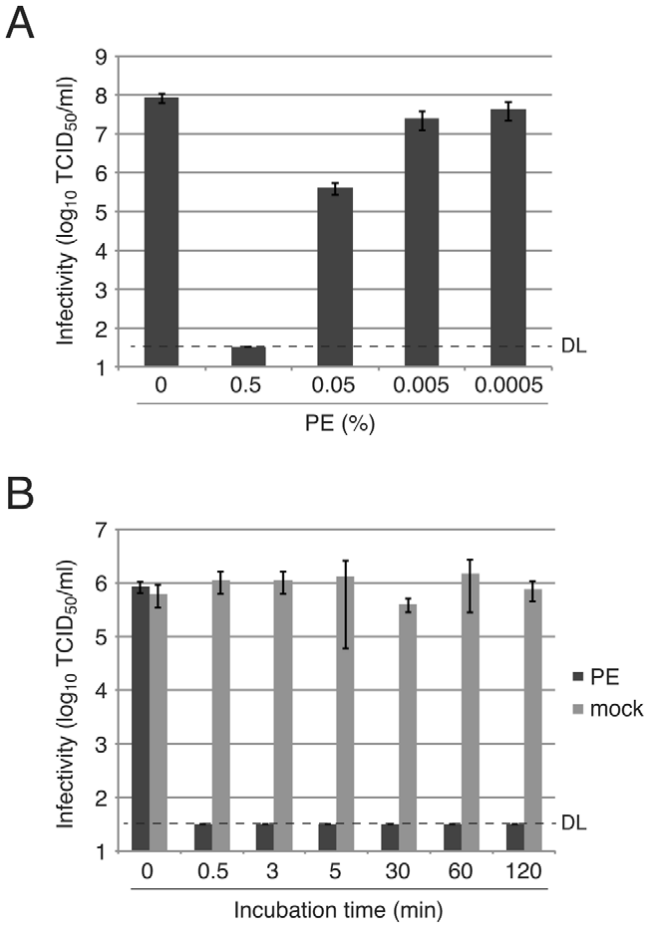
Concentration dependency and time dependency of effects of PE on influenza virus. Influenza virus H3N2 was treated with different concentrations of PE for 3 min (A) or with 0.5% PE for different incubation times (B), followed by measurement of virus infectivity with the standard TCID_50_ assay. PE-treated virus infectivity is plotted in the black bar and mock-treated infectivity is shown in the gray bar. Average infectivities of three independent experiments are shown in the graph. Error bars indicate standard deviations. DL, detection limit of virus infectivity with the method.

We further characterized the anti-virus effect of PE on viruses by using influenza virus H3N2. Dilution of PE revealed that 0.005% PE (1/100 dilution) had lost most of its anti-virus activity (Fig. 3A). Next, we investigated incubation time necessary for virus inactivation. Influenza virus H3N2 and 0.5% PE were mixed, and after 0.5, 3, 5, 60 and 120 min, the mixture was diluted to 100 times with DMEM to stop the reaction. Measurement of infectivity revealed that even 0.5 min (30 sec) is sufficient time to inactivate the virus completely (Fig. 3B), indicating that the anti-virus effect of PE is exerted in a short time. Incubation time 0 means that the virus and PE were individually diluted and cells in a 96-well plate were infected with the diluted virus first, and then the diluted PE was added to the wells.

### Effects of PE in steps of virus infection

**Figure 4:**
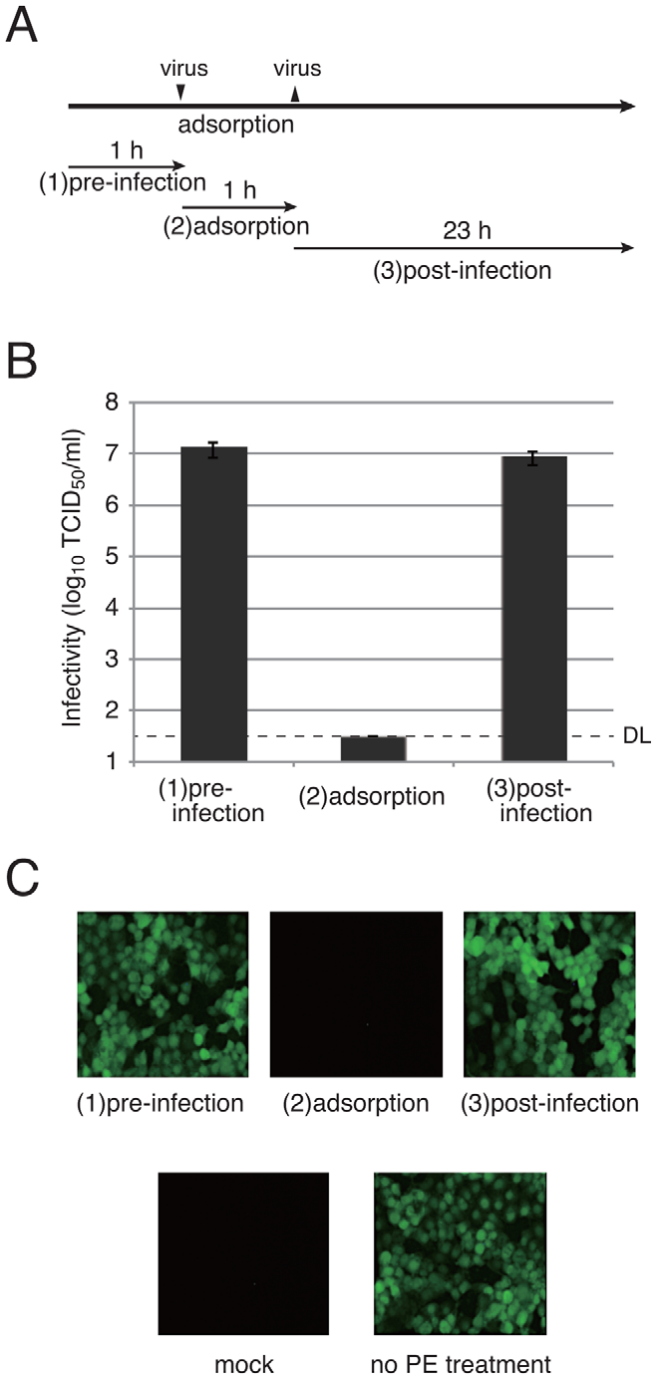
Effects of PE on influenza virus and SeV in different steps of infection. For determining the infectivity of influenza virus H3N2 by the standard TCID_50_ method, MDCK(+) cells were incubated with 0.025% PE before virus infection for 1 h [A, (1)], during virus adsorption for 1 h [A, (2)], or after virus infection for 23 h [A, (3)]. (B) Virus infectivities measured are plotted in a graph. Error bars indicate standard deviations. DL, detection limit of virus infectivity with the method. (C) In SeV-EGFP infection at an input multiplicity of infection of 5, LLC-MK_2_ cells were treated with 0.025% PE as in (A), and GFP fluorescence was observed after 24 h.

To determine the step of virus infection in which PE exerts its effect, PE was included in different steps of influenza virus infection in a standard TCID_50_ assay (Fig. 4A). PE was included in the periods of (1) pre-infection, (2) adsorption, and (3) post-infection. Measurement of virus infectivity demonstrated that PE inactivated influenza virus infection when it was applied in the period of virus adsorption (Fig. 4B). This suggests that PE suppressed virus replication in the step of virus adsorption to cells.

A similar experiment was performed in SeV infection. LLC-MK_2_ cells were infected with SeV possessing the EGFP gene (SeV-EGFP) with PE treatment in the three different conditions described above. After 24 h, EGFP expression was observed under a fluorescent microscope. Fig. 4C indicates that treatment with PE in the period of virus adsorption abolished EGFP production. Treatment of cells with PE before and after infection, however, did not restrict virus infection. In a separate experiment, effects of PE on virus attachment and virus invasion were individually investigated (Fig. S1). Only in the condition that PE was included in virus attachment but not in endocytosis and the membrane fusion, virus replication was restricted almost completely (Fig. S1). These results suggest that PE works by inhibiting virus attachment to cells.

### Hemagglutinating inhibition (HI) by PE

**Figure 5:**
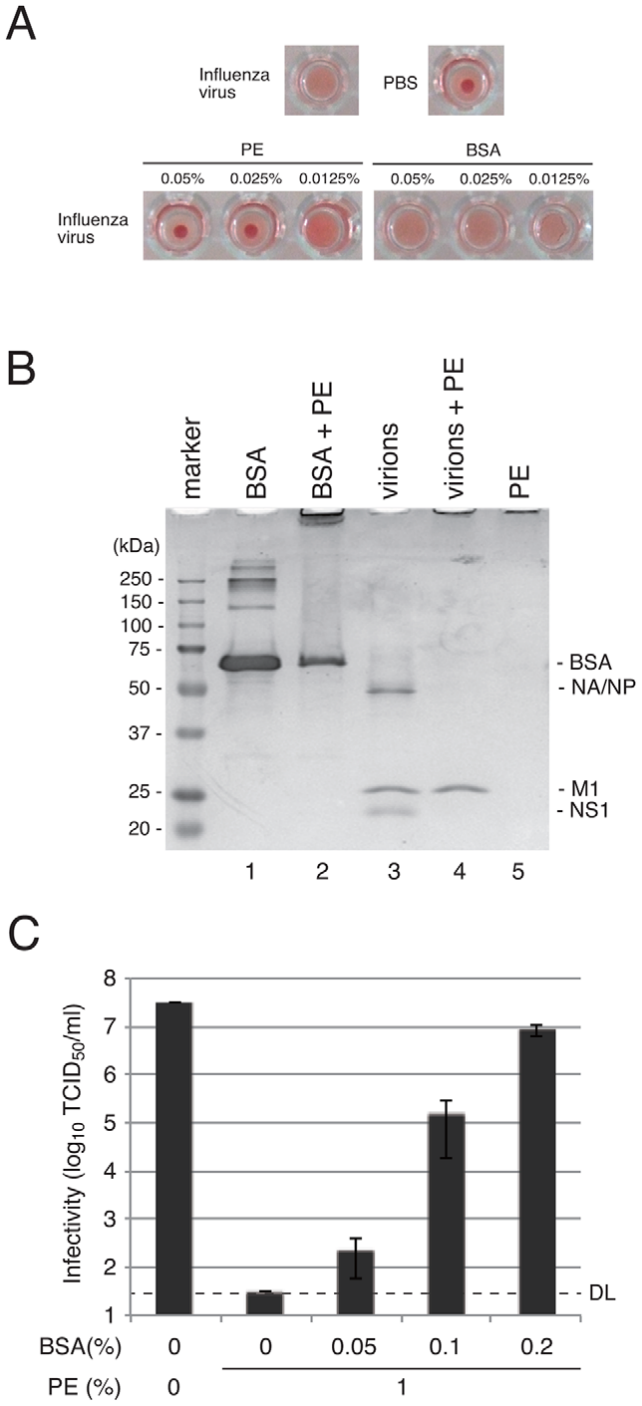
Interaction of PE with influenza virus proteins. (A) Hemagglutination inhibition by PE. Influenza virus H3N2 was reacted with different concentrations of PE in a round-bottom 96-well plate, followed by addition of chicken red blood cells in PBS. Hemagglutination was assessed after 1 h. Bovine serum albumin (BSA) was used instead of PE as a control. (B) Protein aggregation induced by PE. Purified influenza virions or BSA were incubated with PE, and proteins were analyzed by SDS-PAGE and Coomassie brilliant blue staining. Migrating positions of proteins and molecular weights of protein size markers are shown on the sides of the gel panel. (C) Abrogation of PE effects by BSA. Influenza virus and PE were incubated in the presence of different concentrations of BSA. Average infectivities of three independent experiments are shown in the graphs and error bars indicate standard deviations. DL, detection limit of virus infectivity with the method.

To further support the notion that PE inhibits virus attachment to cells, HI by PE was investigated. High concentrations (0.05% and 0.025%) of PE inhibited hemagglutination by influenza virus, while the same concentrations of BSA did not (Fig. 5A). These results indicate that PE restricted attachment of influenza virus to chicken erythrocytes. Interestingly, PE at higher concentration (0.1% or more) caused hemagglutination without influenza virus (data not shown). This suggests that PE at a high concentration directly reacted with chicken red blood cells, resulting in hemagglutination. PE was presumed to bind to proteins on virus particles and the cell surface.

### Effect of PE on virions

We next investigated interaction of PE with virions (virus particles). Purified virions of influenza virus H3N2 were incubated with PE at room temperature for 10 min, and viral proteins were analyzed by SDS-PAGE (Fig. 5B). Viral protein bands disappeared from the original positions, except for the M1 protein band, with PE treatment, but the protein band at the top of the lane became denser (Fig. 5B, lane 4). This band at the top of the lane was slightly denser than that of PE treatment alone (Fig. 5B, lane 5). This result suggested that viral proteins formed large aggregates and were trapped at the well of the gel. Treatment of BSA instead of purified virions with PE caused a similar phenomenon: reduction of the BSA protein band from the original migrating position and appearance of a thick protein band around the top of the gel (Fig. 5B, lane 2). We further investigated aggregation of H3N2 virions by other tannin reagents. WE, GTE, CE, PGG, PRG and PYG did not appear to form protein aggregation in a similar SDS-PAGE analysis, while PE caused aggregation of virus proteins (Fig. S2). Similar results were obtained when SeV particles were used instead of H3N2 virions (data not shown). These results suggest that PE, which has binding capacity with proteins, causes aggregation of proteins.

To confirm this notion, competition of BSA with PE in anti-virus effects was investigated. PE and different concentrations of BSA were incubated, and then the PE solution was subjected to an anti-virus test for influenza virus H3N2 (Fig. 5C). Increasing amounts of BSA caused increasing virus infectivity, and 0.2% BSA almost completely abrogated the anti-virus ability of PE (Fig. 5C). These results suggest that PE exerts if anti-virus effect by binding to viral proteins in virions.

### Effects of PE on virus after accelerated aging

**Figure 6:**
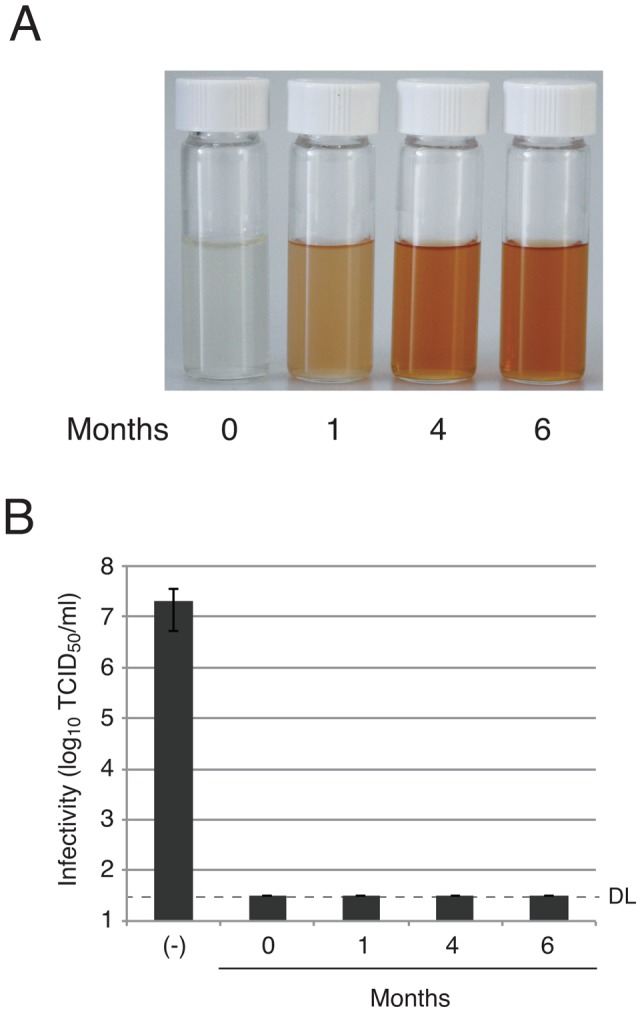
Accelerated aging of PE and its anti-viral effects. (A) 1% PE solution underwent accelerated aging under high temperature and humidity conditions for 6 month. Samples were photographed. (B) Accelerated aged PE samples were diluted to 0.5% and used for anti-virus assay by using influenza virus H3N2. Average infectivities of three independent experiments are shown in the graphs and error bars indicate standard deviations. DL, detection limit of virus infectivity with the method.

Polyphenols are strong anti-oxidants, and oxidation changes at least their color with time [17]. PE gradually undergoes color change by oxidation from clear to brown. To investigate the change in anti-virus effects associated with color change, we prepared aged PE samples. According to a guideline of stability tests of medicine, PE solution was kept under the condition of 40°C and relative moisture of 75% in a dark room for 1, 4, or 6 months. By this procedure, PE solution gradually became brown (Fig. 6A). Anti-virus activity against influenza virus, however, did not change (Fig. 6B). With a 10-times dilution of PE, in which the virus was partially inactivated and a subtle difference was observed (Fig. 3A), there was also no difference in anti-virus effect (data not shown). These results indicate that the anti-virus activity of PE is stable in an accelerated aging test.

## Discussion

It is important for prevention of virus infection to inhibit transmission of pathogens. One of the means is the use of an anti-microbial reagent. However, some viruses, especially non-enveloped viruses such as human norovirus, have high levels of resistance to anti-microbial reagents such as detergents and ethanol. In the present study, we searched for an anti-viral reagent to efficiently inactivate both enveloped and non-enveloped viruses. We investigated plant-derived tannin samples, since catechins, tannins from green tea or black tea, were reported to inhibit pathogenic viruses such as influenza virus, herpes simplex virus type 1 and human immunodeficiency virus [7], [8], [18]–[21]. We employed seven different tannin samples at a non-cytotoxic concentration and investigated their anti-virus effects against six enveloped and six non-enveloped viruses. Since some tannin samples such as PE and WE contain complex constituents, we employed crude extracts for those. The results clearly showed that PE alone inactivated all of the viruses to an undetectable level (Fig. 2, Table 3). We then characterized the inhibition of virus infection by PE mainly by using influenza virus.

When PE was applied to cells before or after infection, it did not suppress virus infection. However, PE abrogated virus infection when it was applied in the period of virus adsorption. PE appeared to react with virus particles and inactivate virus attachment to cells. Inhibition of adsorption was reported in inactivation of herpes simplex virus by tannic acid by using radiolabeled virus particles [19]. On the other hand, the inhibition of HIV-1 infection by epigallocatechin gallate was shown to be due to the binding of the catechin with the CD4 receptor on the cell surface [22], [23]. In the present study, high concentrations of PE (0.1% or more) caused aggregation of chicken blood cells without influenza virus (data not shown). Thus, a high concentration of persimmon tannin may also interact with molecules on the cell surface.

SDS-PAGE analysis showed that PE induced aggregation of purified virions or BSA probably through association with proteins. Epigallocatechin gallate was previously shown to inactivate herpes simplex virus, and SDS-PAGE analysis demonstrated a complex formation of purified viral glycoproteins by the catechin [7]. Not only persimmon tannins but also other tannins have the potential to induce viral protein aggregation. At the concentrations and under the conditions of electrophoresis used in this study, however, only PE induced aggregation. The high ability of protein aggregation of PE may be related to its strong and broad range anti-viral activity.

Green tea extracts (GTE) contain hydrolyzable tannins and the main ingredients are (−)-epicatechin, (−)-epigallocatechin, (−)-epicatechin-3-gallate and (−)-epigallocatechin-3-gallate [24]. Persimmon extracts contain condensed tannins and its main ingredients are epicatechins similar to those of GTE [25], but they condense and form a higher structure [26]–[28]. The molecular weight of persimmon tannin is 13,800 or more, whereas molecular weights of hydrolyzable tannins are thought to be 500–3,000 [6], [28]. It is thus presumed that the ability of interaction of a higher structure of persimmon tannins caused intense binding with proteins and potent anti-virus effects.

In summary, the present study revealed that PE has inhibitory effects on the broadest range of viruses among the seven representative plant-derived tannin preparations. Investigation of the inhibitory mechanism of influenza virus and SeV suggested that persimmon tannins interacted with virion proteins and restricted virus adsorption to the cells. Due to the rapidly elicited anti-viral effect of persimmon tannins and their stability and safety, persimmon tannins can be utilized as anti-virus reagents to prevent virus infection. Especially, PE may be useful for inactivation of norovirus, which is a major cause of foodborne gastroenteritis, on hard surface.

## Supporting Information

Figure S1 Effects of PE on virus attachment and virus invasion were separately investigated. (2a) To investigate the effect of PE treatment on virus attachment, influenza virus H3N2 was mixed with an equal volume of 0.5% PE and incubated for 3 min. After 10-fold serial dilution, 50 µl/well of the diluted virus inoculum was applied to pre-chilled cells and kept at 4°C for 1 h. Cells were then washed with 50 µl/well of pre-chilled PBS three times, incubated in 50 µl of pre-warmed DMEM and kept for 1 h at 37°C. (2b) To investigate the effect of PE treatment on virus invasion, influenza virus H3N2 was applied to pre-chilled cells MDCK cells and kept at 4°C for 1 h. Then the cells were then washed with 50 µl/well of pre-chilled PBS three times and supplemented with pre-chilled DMEM containing PE. The temperature was then increased to 37°C, which causes endocytosis and membrane fusion (virus invasion), and the temperature was maintained at 37°C for 1 h. In both cases, cells were further washed with PBS three times and incubated in 100 μl/well of DMEM containing 20 µg/ml trypsin, followed by infectivity measurement by the TCID_50_ method.
(TIF)

Figure S2 An equal volume of purified H3N2 virions (10 μg/μl) was mixed with a reagent as indicated in the figure and kept for 3 min. The samples were further mixed with an equal volume of 2× SDS sample buffer (0.5 M Tris-HCl [pH 6.8], 10% SDS, 20% glycerol, one grain of bromophenol blue, 1.7 M β-mercaptoethanol) and kept at 50°C for 20 min. Proteins were analyzed by 12% SDS-PAGE and Coomassie brilliant blue staining. Circle indicates proteins probably stacked at the bottom of a loading well of the PE sample.
(TIF)
