# Supplementary figures and images for: Inactivation of Pathogenic Viruses by Plant-Derived Tannins: Strong Effects of Extracts from Persimmon (Diospyros kaki) on a Broad Range of Viruses
Source: PLoS One. 2013 Jan 25;8(1):e55343. doi: 10.1371/journal.pone.0055343 (PMC3555825; doi:10.1371/journal.pone.0055343)

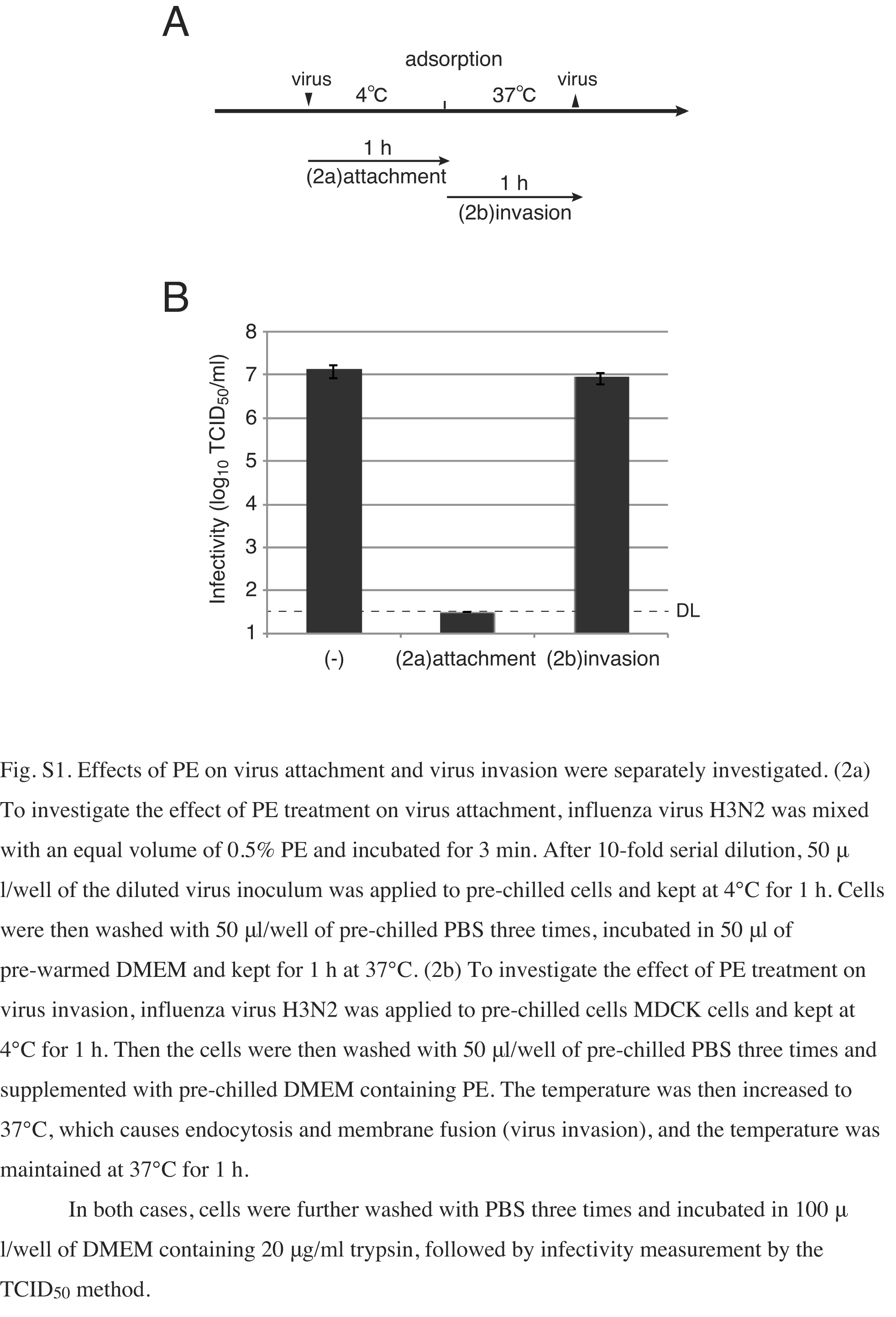

Supplement: Figure S1 — Effects of PE on virus attachment and virus invasion were separately investigated. (2a) To investigate the effect of PE treatment on virus attachment, influenza virus H3N2 was mixed with an equal volume of 0.5% PE and incubated for 3 min. After 10-fold serial dilution, 50 µl/well of the diluted virus inoculum was applied to pre-chilled cells and kept at 4°C for 1 h. Cells were then washed with 50 µl/well of pre-chilled PBS three times, incubated in 50 µl of pre-warmed DMEM and kept for 1 h at 37°C. (2b) To investigate the effect of PE treatment on virus invasion, influenza virus H3N2 was applied to pre-chilled cells MDCK cells and kept at 4°C for 1 h. Then the cells were then washed with 50 µl/well of pre-chilled PBS three times and supplemented with pre-chilled DMEM containing PE. The temperature was then increased to 37°C, which causes endocytosis and membrane fusion (virus invasion), and the temperature was maintained at 37°C for 1 h. In both cases, cells were further washed with PBS three times and incubated in 100 μl/well of DMEM containing 20 µg/ml trypsin, followed by infectivity measurement by the TCID50 method. (TIF) [file pone.0055343.s001.tif]

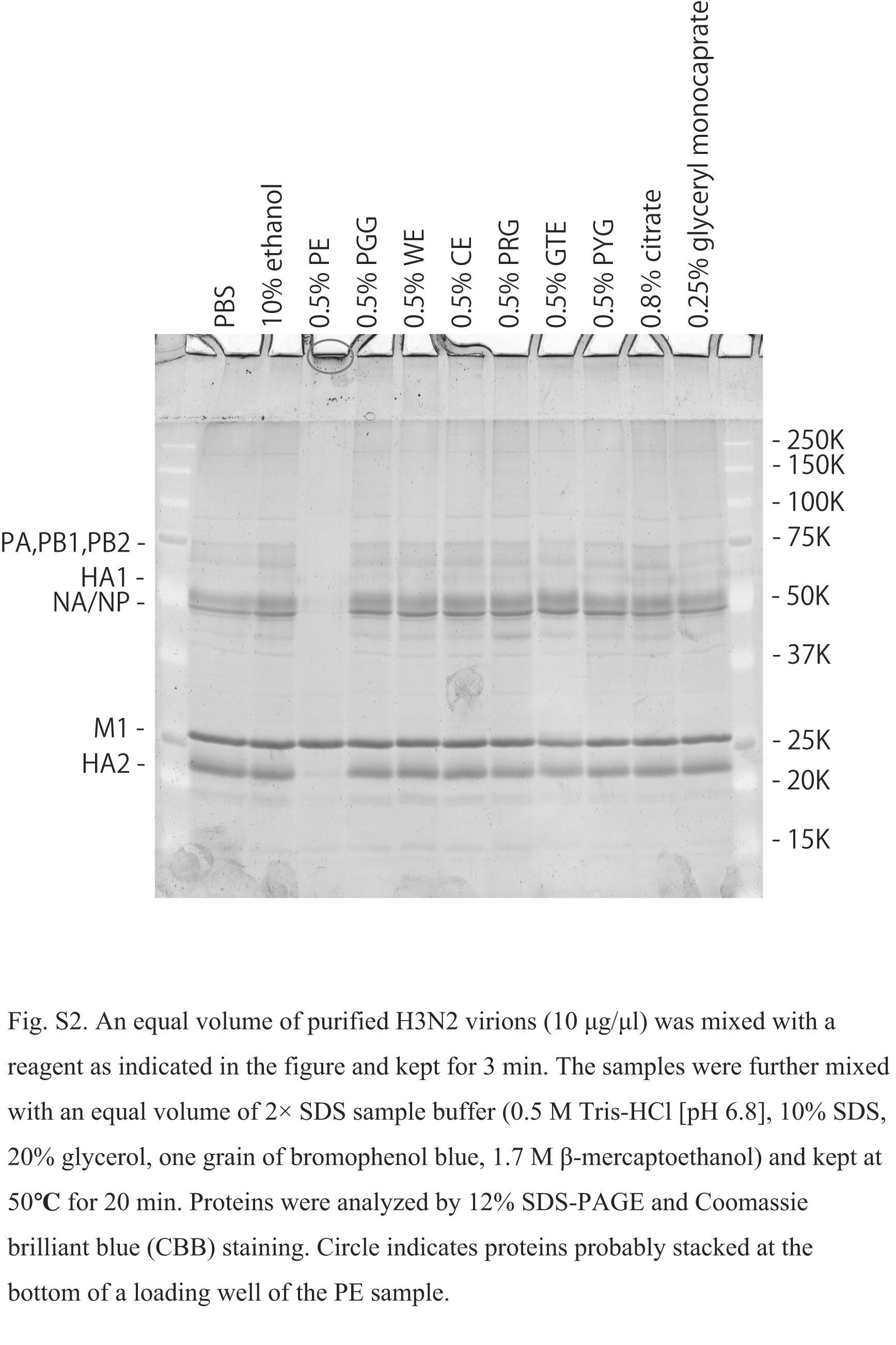

Supplement: Figure S2 — An equal volume of purified H3N2 virions (10 μg/μl) was mixed with a reagent as indicated in the figure and kept for 3 min. The samples were further mixed with an equal volume of 2× SDS sample buffer (0.5 M Tris-HCl [pH 6.8], 10% SDS, 20% glycerol, one grain of bromophenol blue, 1.7 M β-mercaptoethanol) and kept at 50°C for 20 min. Proteins were analyzed by 12% SDS-PAGE and Coomassie brilliant blue staining. Circle indicates proteins probably stacked at the bottom of a loading well of the PE sample. (TIF) [file pone.0055343.s002.tif]
